# Supplementary material for: Patterns of Intron Gain and Loss in Fungi
Source: PLoS Biol. 2004 Nov 30;2(12):e422. doi: 10.1371/journal.pbio.0020422 (PMC532390; doi:10.1371/journal.pbio.0020422)
Supplement: Table S1 — Also available at http://genes.mit.edu/NielsenEtAl/. (4.3 MB ZIP). [file pbio.0020422.st001.zip › NielsenEtAl/html/146.html]

AN4264.1.NCU02811.1.MG01593.1.FG06842.1


```
 CLUSTAL W (1.82) Multiple Sequence Alignments - Introns Inserted


Sequence 1: MG01593.1	498 aa
Sequence 2: FG06842.1	491 aa
Sequence 3: NCU02811.1	526 aa
Sequence 4: AN4264.1	494 aa
Alignment Length: 561 aa
Number Identitical Residues: 179 aa
Alignment Score (without introns) 9984


MG01593.1 	MAFGD~---------------------ERKRPAALNLTPARSGST------DSTSTSSSL
NCU02811.1	MAFND~---------------------EKKRPAALNLTPQRTMSAGSVSSNNSSSTSSSL
FG06842.1 	MAGPE1---------------------DKRRPAALNLTAARPGTS------ESSSSDGSL
AN4264.1  	MSVSS~DSQNDSPQTYEPTFPREFVSSENRRSMASSPQPATVGAQDS----SSESMAAPL
          	*:  . .:...:..: ..: . .  :::.:*. * .  .    : .:    .* *  ..*

MG01593.1 	KN-PRTPRFAEATSIHSPVEDMSR-KPFDD-ERSFQAQSQPGDIGFGYINESSSNRESVT
NCU02811.1	AKPPRTPRFAEATSVHSPVDGRT--LPFSD--QSEVAHAQPGDVGFGYIG-NGGNRESVA
FG06842.1 	K--PRTPRFAEATSVHSPVEARS---PFADPEKSHVAQPQPADIGFGYI----AHRESIP
AN4264.1  	KS-PRSARFAEATAINSPSAAESSRSPFADPPNQSQNAPDVSDVGFGYVN-AQDPTQHVP
          	 . **:.******:::**    :   ** *. ..    .: .*:****:.      : :.

MG01593.1 	MPMTPRSPLKSAMRVPGTPARKFDNPLSPTFREEQVLEKREASTDVEQAKDL0KVKTRVR
NCU02811.1	MPMTPRSPLKSAMKVPGTPGRTLANPLSPTFREEEELEVKEKLTEKEQARDL0KIKTRVR
FG06842.1 	VPMTPKTPLKSAMKVPGTPA-HLRNPMSPTFREEDILEKRELSTEKEQARDV0KIKARVR
AN4264.1  	HHAAPASPLKSALRVPGTPARTLN-PLSPTFREEFNLEKQEKQAEKANAKDL0AIKVRVR
          	   :* :*****::*****.  :  *:*******  ** :*  ::  :*:*:  :*.***

MG01593.1 	MAKFALRGVNFSCSLIILAMISTTFTIFNATKSLPTRDGKPAWSTTTEIWPQILILTVAS
NCU02811.1	MAKFALRGVNFSCSLIILSMLSASFSIFNATRHLPAMNGLPAWAAGTNPWPQYVVLACSC
FG06842.1 	LAKFALRGVNFSCSLIILAMLSASFSIFNATRSLPSQSKMPSWAQNTKAWPQKLVLAMAC
AN4264.1  	VAKIFLRFVSFGCSLIVLTIIATTLTIFHATKNLPPRSGTTPWAVGTNPWPQYLLLATAS
          	:**: ** *.*.****:*::::::::**:**: **. .  ..*:  *: *** ::*: :.

MG01593.1 	ISLLICVCIFWAYCRGGHARAEKVGVYYTLFAVGW~FIFSMVMWAIAAGIFQHKRNSSGN
NCU02811.1	VSLLICIGVFIGYCRGGHQRAEKVGVYYTLFAVGW~FIFSMIMWAVAAGIFQFTKNNSKN
FG06842.1 	VSLLACILVFVAYCRGGHKRAEKVGTYYTMFAIGW0FIASLILWVITAIIFQNSKNNSNN
AN4264.1  	LSLLSCLVVFWTWKKRGYKRAEKVAVYYSIFSVGF~FGFTLILWVVVSAIYQNSKSNGNN
          	:*** *: :*  : : *: *****..**::*::*: *  ::::*.:.: *:* .:... *

MG01593.1 	QDMWGWSCVQ-NHRADLFDEKVDYALLCRLQ0NWTMVCIIIEIVVEVIAITLYSIVFYRY
NCU02811.1	QDMWGWSCVE-NHRAELFKDKVDYALVCRLQ0NWGLICMIIEIIIEIISITLYSIVFYRY
FG06842.1 	KDMWGWSCVN-NHRSDVFSEKVDYALVCRLQ~NWAMICIIIEIVVEVLAILLYSVVFYRY
AN4264.1  	KDLWGWSCVTGNLRSTAYASDIDYPLLCRLQ0DWGLVCAVIEIVIEVLVILIYAVVFYRF
          	:*:****** .* *:  : ..:**.*:**** :* ::* :***::*:: * :*::****:

MG01593.1 	YSKRKLHKSMDLRDKARSDLYLAQLRSQSAPNTPGF--GPKSPAFSQYAMSPRFPPQAYG
NCU02811.1	WSKRKLLKSMNMRDKARSDLYLAQLRVQSAPNTPGF--GPKSPTFSQYALSPRFPPSQYQ
FG06842.1 	YTKRRLFKSMDMRDRARSDLYLAQLRSQSAPNTPGF--GPKSPALSAYAMSPRHPPAAYR
AN4264.1  	WSKRKLAKSMDRRDKARSDLYLAQLRLQSAPNTPGFPLSPKSPWVSTTMQDP------YS
          	::**:* ***: **:*********** *********. .**** .*    .*      * 

MG01593.1 	SLGDIQ----------ETS---PFTPGGRPAPIAEPQSSFAASNSPTQPFKLQAPPLKAP
NCU02811.1	SFDDVEKGYGPGPTQQQQQQQQPTTPGGQ-RLIIPQQSSFSSPKIDTG-FKLQAPPSKAN
FG06842.1 	NLSDIS----------ENPNPNPFTPG---TQFAEPPSQFAPQDTG---FKLQAPPVKAP
AN4264.1  	SAEKGE-----------------ATPT----QFATP----ASPTRPQPTFQLQPPPIRVQ
          	.  . .                  **      :       :.      .*:**.** :. 

MG01593.1 	SATPRAQQNGFESPSTASNHN-----------LTFTPSASASAAPTVDSDEPRYDAVPIP
NCU02811.1	PATPVTPKGGFMTPSSSNAVSPVDPMPQINLPQPPPPAVVQIAHAPMADGEQQYEAVPIP
FG06842.1 	SATPKLNQSAFT-PTDTTPPS-----------LPMVPTVHVSEHGPVGSDEPTYDAVPIP
AN4264.1  	HATPKSEQEEFPASAPTPSPP--------------------QEHMGAAPGERTYEAVPIP
          	 ***   :  * :.: :                                .*  *:*****

MG01593.1 	GAYSDQAVKSPPP--TRTAFQ----
NCU02811.1	GAYAGQAIKNPPPPPMQTRFA----
FG06842.1 	GAYTGQAIKSPPP--AQTSFGQGYY
AN4264.1  	VAYASPMSPSFPQG-AR--------
          	 **:.    . *    :
```
